# Completeness of reporting of randomised controlled trials including people with transient ischaemic attack or stroke: A systematic review

**DOI:** 10.1177/2396987318782783

**Published:** 2018-06-20

**Authors:** Blair Wilson, Peter Burnett, David Moher, Douglas G Altman, Rustam Al-Shahi Salman

**Affiliations:** 1 Medical School, University of Edinburgh, Edinburgh, UK; 2 Edinburgh Royal Infirmary, NHS Lothian, Edinburgh, UK; 3 Centre for Journalology, Clinical Epidemiology Program, Ottawa Hospital Research Institute, Ottawa, Canada; 4 Centre for Statistics in Medicine, University of Oxford, Oxford, UK; 5 Centre for Clinical Brain Sciences, University of Edinburgh, Edinburgh, UK

**Keywords:** Stroke, reporting, Consolidated Standards Of Reporting Trials, randomised controlled trial

## Abstract

**Purpose:**

To assess the adherence of stroke randomised controlled trials to Consolidated Standards Of Reporting Trials reporting guidelines and investigate the factors that are associated with completeness of reporting.

**Method:**

We took a random sample from the Cochrane Stroke Group's Trial Register of transient ischaemic attack or stroke randomised controlled trials, published in English in 1997–2016 inclusive. Two reviewers assessed the published report of the final primary results of stroke randomised controlled trials with a 10-point truncated Consolidated Standards Of Reporting Trials reporting checklist to investigate adherence over time, univariable associations and independent associations with total Consolidated Standards Of Reporting Trials reporting score in a multiple linear regression model.

**Findings:**

In this random sample of 177 stroke randomised controlled trials, the mean score on the truncated Consolidated Standards Of Reporting Trials checklist was 5.8 (SD 2.2); reporting improved from 1997–2000 (4.9 SD 2.0) to 2001–2009 (5.8 SD 2.1) and to 2010–2016 (6.8 SD 2.1). A higher Consolidated Standards Of Reporting Trials score was independently associated with publication during epochs following a revision of Consolidated Standards Of Reporting Trials reporting guidelines (p < 0.001), journal endorsement of the Consolidated Standards Of Reporting Trials reporting guideline at the time of randomised controlled trial publication (p < 0.001) and modified journal impact factor using median citation distribution (p = 0.012).

**Discussion:**

Stroke randomised controlled trial reporting to Consolidated Standards Of Reporting Trials standards has improved over time, but could be better.

**Conclusion:**

Journal endorsement and enforcement of Consolidated Standards Of Reporting Trials reporting guidelines could further improve the reporting of stroke randomised controlled trials.

**Systematic review registration:** Registered with PROSPERO (CRD42017072193).

## Introduction

Randomised controlled trials (RCTs) are the fairest tests of treatment, but judgements about their value are dependent on transparent and complete reporting. Inadequate reporting prevents a complete assessment of an RCT’s risk of bias and description of their results, which can also preclude the re-use of data in meta-analyses.^1,2^ In order to combat this, the Consolidated Standards Of Reporting Trials (CONSORT) Statement was developed in 1996,^3^ updated in 2001^4^ and revised in 2010^5^ to improve the reporting of RCTs. The CONSORT guidelines seem to have been endorsed by over 600 journals.^6^

In general, studies of RCT reporting have not only demonstrated incomplete reporting in numerous specialties but also modest improvements over time that were often associated with journal endorsement and uptake of CONSORT.^7–20^ Concerns about completeness of reporting remain, particularly in journals with low impact factors.^7,21^

Despite advances in the prevention and treatment of stroke supported by RCTs, stroke remains the leading cause of disability and second leading cause of death worldwide and stroke burden is projected to increase with changes in lifestyle and longevity.^22,23^ The limited funding available for stroke research to lessen this burden should not be wasted.^24,25^ But concerns remain about waste in stroke research, including the poor reporting of research.^26,27^

Other than an investigation of the reporting of a specific intervention for stroke rehabilitation,^28^ the last systematic assessment of stroke RCT reporting pre-dated CONSORT.^9^ That review found that the standard of reporting was poor, but it improved over time alongside an increase in RCT sample size. Reporting did not appear to be associated with journal impact factor but trials with a positive outcome tended to be less well reported than those with neutral or negative outcomes.^9^ This differs from the findings in other specialties.^14,15^ However, it is unclear whether stroke RCT reporting has improved since CONSORT guidelines were released and updated, what factors are associated with better reporting, and whether these associations differ from other diseases.^27^

Therefore, we aimed to assess: the extent to which the published reports of the final primary results of RCTs involving participants with transient ischaemic attack (TIA) or stroke have adhered to the CONSORT reporting guidelines between 1997 and 2016; whether adherence has changed over time, in particular following each revision of the CONSORT guidelines; and which factors are associated with better reporting.

## Methods

### Protocol and registration

All authors developed and approved the protocol, which we registered with PROSPERO before embarking on data collection (CRD42017072193).

### Eligibility criteria

We included published reports of the final primary results of RCTs including patients with TIA or stroke, published in 1997–2016 inclusive. We applied eligibility criteria designed to obtain a representative sample of RCTs (Table 1).

**Table 1.** Eligibility criteria for included studies.

Inclusion criteria	Exclusion criteria
Published report of the final primary results of an RCT, published in 1997–2016 inclusive English language publication Participants included after TIA or any type of stroke Any type of therapeutic intervention (drug, surgery, device, rehabilitation, etc.)	Reports of interim analyses that preceded the report of the final primary results of an RCT Reports of secondary or long-term follow-up analyses of an RCT Duplicate reports of the final primary results of an RCT Cost-effectiveness and economic studies of an RCT RCTs in which stroke was not the qualifying condition, but the outcome

RCT: randomised controlled trial.

### Information sources

On 30 August 2017, the Managing Editor of the Cochrane Stroke Group searched the Cochrane Stroke Group’s Trial Register for all publications of RCTs including patients with TIA or any type of stroke published in 1997–2016 inclusive.

#### Study selection

We sub-divided the results of the search into three epochs of publication (1997–2000, 2001–2009 or 2010–2016), each corresponding to the timing of a published revision of the CONSORT guidelines.^3,5,29^ We used a random number generator in Microsoft Excel to take a random sample of equal size from each of these three groups of RCTs, removed duplicate records of the same RCT in order to include only the report of the final primary results of each RCT, and took further random samples as required to achieve our target sample size of 180 RCTs. We chose this sample size so that it would adequately power a multiple regression analysis including 10 covariates based on the likely distribution of CONSORT reporting scores and be feasible for two reviewers to assess in the time available to the research team.

### Data collection

We imported the results of the search into Covidence (www.covidence.org). One reviewer (BW) screened the titles and abstracts of all RCTs to exclude any ineligible RCTs. Two reviewers (PB and BW) reviewed the full text of potentially eligible RCTs, independent of each other. PB and BW used the data collection tool within Covidence to independently assess the completeness of reporting of included RCTs. Any protocols that were referenced within the included papers were checked and included in the scoring. PB and BW used Microsoft Excel to extract information from each RCT on pre-specified covariates that we hypothesised might be associated with completeness of reporting. Any uncertainties or disagreements about eligibility, completeness of reporting, or covariates were resolved by discussion with another reviewer (RA-SS).

### Data items

Our primary outcome was a truncated version of the CONSORT checklist comprising the 10 most important CONSORT checklist items, identified by a group of experts from within the CONSORT group, based on their professional opinion and supported by empirical evidence where available (Table 2).^30^ We tackled the problem of partial reporting by adapting the wording of some criteria so that each item could be scored 1 if it was reported or 0 if it was not reported, for a total score ranging from 0 to 10.

**Table 2.** ***C***hecklist items used for the truncated CONSORT score.

Criterion	Description
Outcomes	Explicitly defined, pre-specified, primary outcome measure, including how and when they were assessed
Sample size	Justification for sample size
Sequence generation	Methods used to generate random allocation sequence
Allocation concealment	Explicitly state mechanism used to implement random allocation sequence (such as sealed envelopes or electronic sequence generation, and block sizes) and describe any steps taken to conceal the sequence until interventions were assigned (such as opaque nature of envelopes or a central telephone/web allocation centre). A description of both of these aspects was required to score a point. Where electronic sequence generation was used it had to be clear that this was concealed from researchers, and not predictable, either with a statement or example describing the use of a central allocation centre
Blinding	Clear statement about whether or not anyone (for example, participants’ care providers or those assessing outcomes) was blinded to interventions after assignment
Outcome estimation	For the primary outcome (identified as above), results for each group, the estimated effect size and its precision (i.e. 95% CI)
Harms	Mentions any harms or unintended effects in each group, or statement of no adverse effects
Registration	Registration number and the trial registry
Protocol	Where the trial protocol can be accessed, if available
Funding	Sources of funding and other support (such as supply of drugs) and role of funders

CONSORT: Consolidated Standards Of Reporting Trials.

In our protocol, we specified the following covariates to investigate associations with completeness of reporting based on prior evidence of their association with completeness of reporting in other diseases: (1) CONSORT endorsement by the journal preceding the publication of the RCT (we established this by searching the CONSORT database online, contacting the journal or searching the journal’s archived guidelines);^11–13,20^ (2) year of publication;^7–10^ (3) sample size of the RCT;^8,9,14,21^ (4) number of recruiting sites (single vs. multicentre);^8,9,14,21^ (5) direction and statistical significance of results with reference to aims/hypothesis (positive, neutral or negative);^9,14,15^ (6) type of intervention (drug, surgical or other);^15,21^ (7) funding source (academic/governmental/charitable vs. commercial vs. other)^14,21^ and (8) journal impact factor.^7,8,21^ However, we did not use journal impact factor because it is widely acknowledged to be a flawed metric as it is the arithmetic mean of a highly skewed distribution of citations and it is quoted to a higher level of precision (three decimal places) than is warranted by the underlying data,^31^ and hence we used a ‘modified journal impact factor’ that uses the median – rather than the mean – number of citations.^32^ We also pre-specified (9) TIA/stroke type and (10) intervention type (acute, prevention, rehabilitation), which we hypothesised might influence the completeness of reporting of stroke RCTs.

### Risk of bias

We reduced bias in our assessments of completeness of reporting and covariates of interest by two reviewers assessing them independently. We looked for evidence of differences between reviewers, which might be systematic, by calculating the kappa statistic to assess the inter-reviewer variability for each CONSORT checklist item (Table 2).

### Summary measure

We used the truncated CONSORT score, which was the sum of the score of each of the 10 fields in the truncated list (Table 2) as our summary measure.

### Statistical analysis

We assessed trends in trial characteristics with time using the Cochran–Armitage test for trend. We quantified the mean and SD of the truncated CONSORT score. We assessed associations of categorical covariates with truncated CONSORT score using ANOVA and univariable associations with continuous variables using Spearman’s rank-order correlation. We assessed trends in reporting of individual CONSORT items with time using the Cochran–Armitage test for trend. We entered all 10 covariates into a single multiple linear regression model to investigate the association of each of our covariates with total truncated CONSORT score, after checking linearity of relationships, multivariable normality, multi-collinearity, the absence of auto-correlation and homoscedasticity. We used IBM SPSS 24, with α = 0.05.

## Results

### Study selection

**Figure 1.**
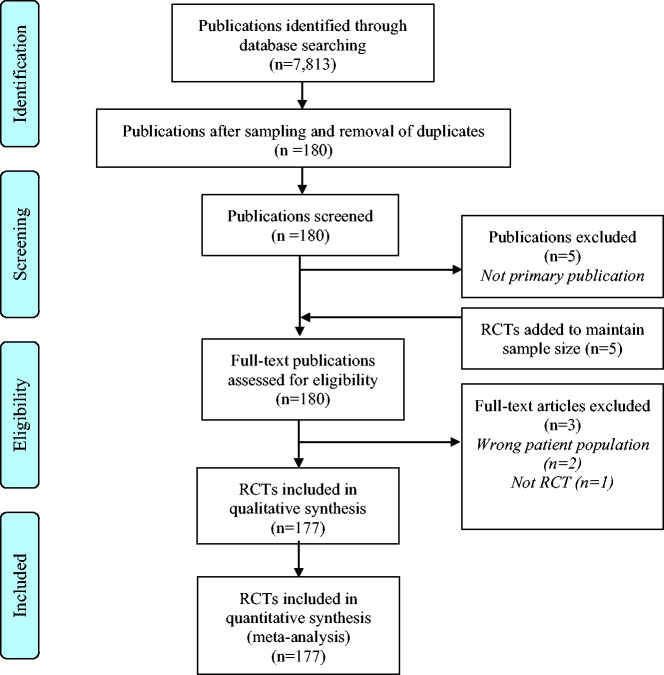
PRISMA flow chart.

From 7813 studies in the Cochrane Stroke Group Trial Register published in 1997–2016 inclusive, we randomly sampled 180 that appeared eligible and included 177 (Figure 1).

### Characteristics of the included RCTs

The RCTs included a variety of combinations of TIA or stroke sub-types, the most frequent being ischaemic stroke alone (Table 3). Three-quarters of RCTs evaluated acute interventions and almost two-thirds of the interventions were drugs. Sample sizes ranged from 8 to 21,106 patients, median 99 (inter-quartile range 41–367). Roughly half of included RCTs reported statistically significant beneficial (i.e. ‘positive’) effects on their primary outcomes. Roughly one-fifth of included RCTs received commercial funding. More than one-third of the journals had not explicitly endorsed the CONSORT statement to require complete RCT reporting to the CONSORT standard. There was a statistically significant downward trend in the proportion of acute trials (p < 0.001), those investigating a pharmacological intervention (p = 0.002) and journals endorsing CONSORT (p = 0.014) over time (the latter due to an increase in the number of open access journals in recent times).

**Table 3.** Descriptive characteristics of included RCTs.

	All 177 RCTs (%)	1997–2000,n = 59 (%)	2001–2009,n = 59 (%)	2010–2016,n = 59 (%)
Type of TIA/stroke included				
Any type	3 (2)	3 (5)	0 (0)	0 (0)
Intracerebral haemorrhage	20 (11)	5 (8)	6 (10)	7 (12)
Ischaemic stroke	84 (48)	30 (51)	29 (49)	25 (42)
Sub-arachnoid haemorrhage	46 (26)	16 (27)	15 (25)	15 (25)
TIA	6 (3)	0 (0)	1 (2)	6 (10)
TIA or ischaemic stroke	12 (7)	3 (5)	6 (10)	3 (5)
Unknown	5 (3)	2 (3)	1 (2)	2 (3)
Type of RCT				
Acute	131 (74)	53 (90)	45 (76)	35 (59)
Prevention	10 (6)	0 (0)	2 (3)	8 (14)
Rehabilitation	32 (18)	6 (10)	10 (17)	16 (27)
Other	4 (2)	0 (0)	2 (3)	0 (0)
Type of intervention				
Drug	112 (63)	47 (80)	34 (58)	31 (53)
Surgical	44 (25)	6 (10)	10 (17)	5 (8)
Other	21 (12)	6 (10)	15 (25)	23 (39)
Number of recruiting sites				
Multicentre	87 (49)	32 (54)	26 (44)	29 (49)
Single centre	82 (46)	25 (42)	30 (51)	27 (46)
Unknown	8 (5)	2 (3)	3 (5)	3 (51)
Sample size^a^				
Median (IQR)	99 (41–367)	142 (32–407)	90 (40–365)	94 (50–233)
RCT outcome				
Positive (p < 0.05)	92 (52)	26 (44)	28 (47)	27 (46)
Neutral	78 (44)	30 (51)	27 (46)	30 (51)
Negative	7 (4)	2 (3)	4 (7)	2 (3)
Funding source				
Not specified	66 (37)	24 (41)	26 (44)	16 (27)
Commercial	35 (20)	16 (27)	11(19)	8 (14)
Other	76 (43)	19 (32)	22 (37)	35 (59)
Journal endorsed CONSORT				
Endorsed	108 (61)	43 (73)	35 (59)	30 (51)
Impact factor^a^				
Median (IQR)	4.2 (2.0–6.0)	4.8 (1.4–6.0)	5.2 (2.1–5.9)	3.0 (2.0–6.2)
Modified impact factor^a,b^				
Median (IQR)	2 (1–5)	2 (1–5)	3 (1–5)	2 (1–5)

TIA: transient ischaemic attack; IQR: inter-quartile range; RCT: randomised controlled trial; CONSORT: Consolidated Standards Of Reporting Trials.

^a^Items are reported as frequency (proportion) for categorical variables, unless otherwise specified for continuous variables.

^b^Modified impact factor is the impact factor for the journal at the time of publication calculated using the median rather than mean of the citation distribution.

### Inter-reviewer agreement

For all 10 items in the truncated CONSORT checklist, inter-reviewer agreement was high, ranging from κ = 0.96 to 1.00 for individual items (online appendix).

### Completeness of reporting

**Figure 2.**
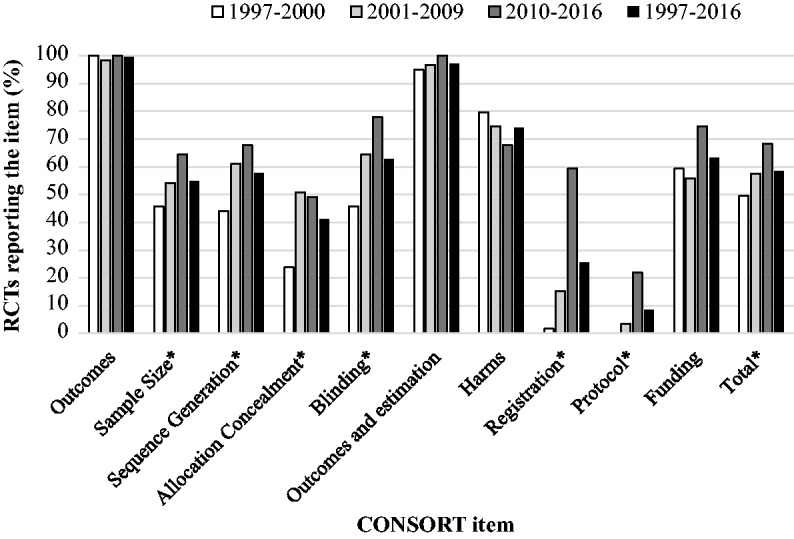
Reporting of individual truncated CONSORT checklist items. From left to right: p=0.042, 0.009, 0.005, <0.001, <0.001, <0.001 and 0.040. *Cochran–Armitage test shows a trend of improvement with time.

In all 177 RCTs, the mean total truncated CONSORT score was 5.8 (SD 2.2) out of 10, (ranging from 1 to 10 in individual RCTs). Completeness of reporting of each of the CONSORT items varied considerably (Figure 2). Explicit definitions of the primary outcome measure and the estimated treatment effect size and its precision were most frequently reported and did not decrease over time. Details of trial registration and the availability of the protocol were least frequently reported, but like many other items (other than harms and funding) there was a statistically significant trend of increasing completeness of reporting individual items over time.

### Associations with completeness of reporting

**Figure 3.**
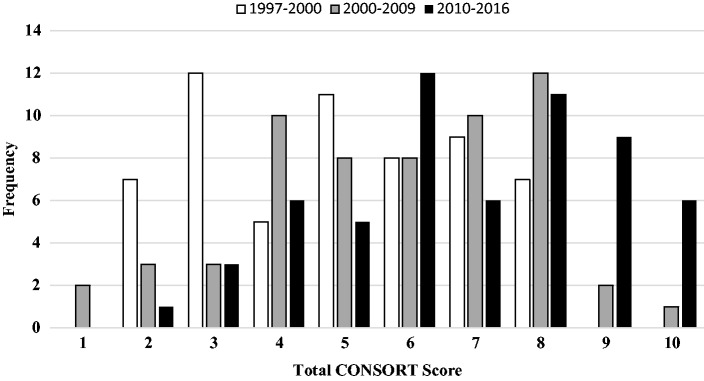
Distribution of CONOSRT Scores by Epoch.

In univariable analyses, there was a significant improvement in total CONSORT score by 1.9 (95% Confidence Interval (CI) 1.0–2.8) from 1997–2000 until 2010–2016 (p < 0.001) and by 1.1 (95% CI 0.2–2.0) from 2001–2009 until 2010–2016 (p = 0.013) (Table 4 and Figure 3). Journal endorsement of CONSORT at the time of an RCT’s publication, higher modified journal impact factor, having a commercial funding source, multicentre recruitment and larger sample size were all associated with higher total CONSORT reporting scores (Table 4). In multivariable analysis, publication during epochs following a revision of CONSORT reporting guidelines was independently associated with higher completeness of reporting (Table 5), as was journal endorsement of the CONSORT reporting guideline at the time of RCT publication, and journal modified impact factor.

**Table 4.** Univariable analyses of associations with truncated CONSORT score.

Categorical covariates	Number of RCTs	Mean total CONSORT score (standard deviation)	p
Year of publication			<0.001
1997–2000	59	4.9 (2.0)	
2001–2009	59	5.8 (2.1)	
2010–2016	59	6.8 (2.1)	
TIA/stroke type^a^			0.28
Haemorrhagic (ICH or SAH)	66	6.1 (2.1)	
Ischaemic (all other groups)	111	5.7 (2.3)	
Trial type			0.11
Acute	46	5.4 (2.3)	
Other	131	6.0 (2.1)	
Type of intervention			0.30
Drug	112	6.0 (2.2)	
Other	65	5.6 (2.3)	
Number of recruiting sites			<0.001
Multicentre	87	6.6 (2.0)	
Single centre/not specified	90	5.1 (2.1)	
Outcome estimate			0.43
Positive	92	5.7 (2.3)	
Negative or neutral	85	6.0 (2.1)	
Funding source			0.022
Purely commercial	35	6.6 (1. 7)	
Other/not specified	142	5.7 (2.3)	
Journal endorsed CONSORT			<0.001
Endorsed	108	6.6 (2.0)	
Not endorsed	69	4.7 (2.0)	
Continuous covariates	Spearman rank-order coefficient		p
Modified impact factor	r_s_ = 0.51		<0.001
Sample size	r_s_ = 0.38		<0.001

RCTs: randomised controlled trials; CONSORT: Consolidated Standards Of Reporting Trials.

^a^Haemorrhagic sub-category included intracerebral haemorrhage and subarachnoid haemorrhage, ischaemic encompassed all other sub-categories as this was the most common sub-type within them.

**Table 5.** Multivariable linear regression analysis of associations with truncated CONSORT score.

		Mean CONSORT score (SD)	Multiple linear regression		
	RCTs (n)		β Coefficient	95% CI	p
Year of publication					
1997–2000	59	4.9 (2.0)	Ref		
2001–2009	59	5.8 (2.1)	1.071	0.435 to 1.709	0.001
2010–2016	59	6.8 (2.1)	2.248	1.559 to 2.937	<0.001
TIA/stroke type^a^					
Haemorrhagic	66	6.1 (2.1)	Ref		
Ischaemic	111	5.7 (2.3)	−0.204	−0.794 to 0.387	0.497
Trial type					
Other	131		Ref		
Acute	46		0.118	−0.626 to 0.863	0.754
Type of intervention					
Other	65	5.4 (2.3)	Ref		
Drug	112	6.0 (2.1)	0.098	−0.505 to 0.702	0.748
Number of recruiting sites					
Other	90	6.6 (2.0)	Ref		
Multicentre	87	5.1 (2.1)	0.672	0.132 to 1.211	0.015
Sample size					
Per n=100 increase	177		0.009	−0.001 to 0.019	0.066
Outcome estimate					
Negative or neutral	85	5.7 (2.3)	Ref		
Positive	92	6.0 (2.1)	−0.216	−0.722 to 0.290	0.400
Funding source					
Other	142	6.6 (1. 7)	Ref		
Purely commercial	35	5.7 (2.3)	0.543	−0.127 to 1.212	0.112
Journal endorsed CONSORT					
Not endorsed	69	6.6 (2.0)	Ref		
Endorsed	108	4.7 (2.0)	1.382	0.726 to 2.038	<0.001
Modified impact factor					
For each unit increase	177		0.127	0.028 to 0.226	0.012

RCTs: randomised controlled trials; CONSORT: Consolidated Standards Of Reporting Trials.

Multiple linear regression model adjusted for year of publication, TIA/stroke type, trial type, type of intervention, number of recruiting sites, sample size, outcome estimate, funding source, CONSORT endorsement and modified impact factor. ‘Ref' indicates which categories were used as reference categories in the multiple linear regression.

## Discussion

In this systematic review of 177 stroke RCTs published over a 20-year period, we found that stroke RCTs on average have reported ∼6 out of 10 items on a truncated CONSORT checklist (Figure 2). Encouragingly, the overall completeness of reporting has increased with time such that stroke RCTs published after the 2010 revision of CONSORT reported ∼7 out of 10 items (Table 4). This improvement may reflect, at least in part, the awareness or endorsement of CONSORT guidelines by the International Committee of Medical Journal Editors in 2001 and individual journals since then.

The reporting of primary outcomes and estimates of treatment effect on these outcomes have been consistently good, while the least well-reported items were the method of allocation concealment, trial registration and protocol location (Figure 2), perhaps because repositories for the latter two were not available in the earlier epochs in this study.^9,28^ This is similar to findings for trials in other diseases,^8,11,16,21,28^ although the proportion of stroke RCTs adequately reporting registration and the location of protocol is particularly low. Each item that was poorly reported in 1997–2000, excluding harms and funding, has shown an improvement in completeness of reporting with time. The reporting of harms appears to have decreased over time, although this might be confounded by the decrease in the proportion of stroke RCTs investigating drug or surgical interventions over time (Table 3), in which the reporting of harms is more of a requirement than for other interventions (e.g. rehabilitation).

We did not find that the type of intervention was associated with completeness of reporting in stroke RCTs in contrast to previous studies in other sub-specialties.^15^ The development and implementation of extensions to the CONSORT statement, addressing the weaknesses in reporting of non-drug interventions, may be responsible for this difference.^33^ Similar to the results of others, this study has shown that the time period of publication,^7–10,14,15^ journal endorsement of CONSORT at the time of publication,^11–13,20^ and multicentre recruitment^21^ are all independently associated with a higher completeness of reporting in stroke RCTs. Others have also identified commercial funding as being associated with better completeness of reporting.^4,34,35^ Our study used a modified journal impact factor, which was associated with a higher total CONSORT checklist score. This finding is in agreement with those of recent studies,^7,8,21^ but in contrast to the earlier stroke RCT study.^9^ We speculate that this could be explained by the increased scrutiny and stricter peer-review processes implemented by higher impact journals in recent years, influenced by the drive to improve completeness of reporting nowadays.^2^ Our study differs from the results of the only previous study of the completeness of reporting of stroke RCTs, which found that completeness of reporting was associated with estimates of treatment effect.^9^

As far as we are aware this is the only study to assess completeness of reporting and the associated factors for stroke RCTs in the 21st century. However, it is not without its weaknesses. We scored RCTs using a modified, truncated CONSORT checklist, to give a score out of 10 as a measure of completeness of reporting of key items. Each of the factors was weighted equally, but these factors vary in their importance; however, any attempt to implement a weighted system to this list would be arbitrary and introduce a degree of subjectivity which would limit the generalisability of our results. An advantage of this binary scoring system was the high inter-reviewer agreement in the assessment of reporting. Other scoring systems are subject to lower and more variable kappa values, e.g. ranging from 0.02 to 0.92.^8^ Lastly, some of our findings may be confounded by factors relating to publication culture: we found that lower impact factor journals exhibited poorer reporting of RCTs, possibly reflecting the fact that higher quality stroke RCTs may be first submitted to higher impact factor journals.

In summary, the standard of reporting of stroke RCTs has improved with time, but there is room for improvement, particularly in lower impact factor journals. This study provides evidence for areas which can be improved. Authors of stroke RCTs should focus on better reporting of the method of allocation concealment, trial registration and protocol availability. The independent associations that we found between journal-level covariates and completeness of stroke RCT reporting suggest that journals may be best placed to improve reporting completeness by endorsement and enforcement of the CONSORT checklist. One study, but not all, shows that making adherence to CONSORT guidelines mandatory improves completeness of reporting.^36^ We therefore suggest that journals require the submission of a completed CONSORT checklist with stroke RCT manuscripts, and that this becomes an integrated part of the peer-review assessment. This may help make reporting standards uniform across journals, and therefore rectify the disparity between journals of high- and low-impact factors. Continuous monitoring of reporting completeness^2^ and other sources of research waste^27^ will be necessary and can be done by researchers in collaboration with the REWARD Alliance (http://rewardalliance.net).

## Supplemental Material

Supplemental material for Completeness of reporting of randomised controlled trials including people with transient ischaemic attack or stroke: A systematic review Click here for additional data file. Supplemental material for Completeness of reporting of randomised controlled trials including people with transient ischaemic attack or stroke: A systematic review by Blair Wilson, Peter Burnett, David Moher, Douglas G Altman and Rustam Al-Shahi Salman in European Stroke Journal
